# Novel Carboxylic Acid-Capped Silver Nanoparticles as Antimicrobial and Colorimetric Sensing Agents

**DOI:** 10.3390/molecules27113363

**Published:** 2022-05-24

**Authors:** Muhammad Imran Irfan, Fareeha Amjad, Azhar Abbas, Muhammad Fayyaz ur Rehman, Fariha Kanwal, Muhammad Saeed, Sami Ullah, Changrui Lu

**Affiliations:** 1 Department of Chemistry, Chemical Engineering and Biotechnology, Donghua University, Shanghai 201620, China; imran.irfan@uos.edu.pk; 2 Institute of Chemistry, Faculty of Science, University of Sargodha, Sargodha 40100, Pakistan; fareeha351@gmail.com (F.A.); samichemist30@gmail.com (S.U.); 3 Department of Chemistry, Government Ambala Muslim Graduate College, Sargodha 40100, Pakistan; 4 Med-X Research Institute, School of Biomedical Engineering, Shanghai Jiao Tong University, Shanghai 201620, China; farihakaanwal@gmail.com; 5 Department of Chemistry and Chemical Engineering, SBA School of Science and Engineering, Lahore University of Management Sciences (LUMS), Lahore 54792, Pakistan; muhammad.saeed@lums.edu.pk

**Keywords:** AgNPs@AA, colorimetric sensing, mercury, nanoparticles, antimicrobials, XDR typhoid

## Abstract

The present work reports the synthesis, characterization, and antimicrobial activities of adipic acid-capped silver nanoparticles (AgNPs@AA) and their utilization for selective detection of Hg^2+^ ions in an aqueous solution. The AgNPs were synthesized by the reduction of Ag^+^ ions with NaBH_4_ followed by capping with adipic acid. Characterization of as-synthesized AgNPs@AA was carried out by different techniques, including UV–Visible spectroscopy, Fourier Transform Infrared Spectroscopy (FTIR), Scanning Electron Microscopy (SEM), X-ray diffraction (XRD), Dynamic Light Scattering (DLS), and zeta potential (ZP). In the UV–Vis absorption spectrum, the characteristic absorption band for AgNPs was observed at 404 nm. The hydrodynamic size of as-synthesized AgNPs was found to be 30 ± 5.0 nm. ZP values (−35.5 ± 2.4 mV) showed that NPs possessed a negative charge due to carboxylate ions and were electrostatically stabilized. The AgNPs show potential antimicrobial activity against clinically isolated pathogens. These AgNPs were found to be selectively interacting with Hg^2+^ in an aqueous solution at various concentrations. A calibration curve was constructed by plotting concentration as abscissa and absorbance ratio (A_Control_ − A_Hg_/A_Control_) as ordinate. The linear range and limit of detection (LOD) of Hg^2+^ were 0.6–1.6 μM and 0.12 μM, respectively. A rapid response time of 4 min was found for the detection of Hg^2+^ by the nano-probe. The effect of pH and temperature on the detection of Hg^2+^ was also investigated. The nano-probe was successfully applied for the detection of Hg^2+^ from tap and river water

## 1. Introduction

Heavy metals are highly toxic to humans in high concentrations and cause harmful effects on the respiratory, renal, and nervous systems [1,2]. These metals may sneak into the drinking and groundwater from industrial wastes and remain persistent in the ecosystem [3,4]. Even at non-hazardous levels, heavy metals may cause accumulative toxicity. The sensing and removal of heavy metals are required to avoid their harmful effects on humans and the environment [5]. Being a heavy metal, mercury has become a serious threat to environmental and living organisms. The primary sources of environmental mercury include industrial wastes, mining operations, and coal-burning power plants [6,7]. In its elemental form, mercury can cross the lipid bilayer and enter living cells [8]. Various analytical methods have been used for the detection of mercury ions—e.g., electrochemical [9,10,11], fluorescence [12,13,14], and colorimetric methods [15,16]. The colorimetric methods are relatively cheaper, with high sensitivity and accuracy representing a change in concentration of the analyte by a color change. A sensitive colorimetric method for the visual detection of mercury ions can be preferred being fast, cheaper, and sensitive. Previously, silver and gold nanoparticles were advantageous for rapidly detecting mercury ions in environmental samples due to changes in color upon aggregation [14,17].

Since the last century, nanotechnology has emerged as a well-known emerging field of science [18,19,20], where nanoparticles (NPs) make an extensive class of materials used in electrochemical [21,22,23] and colorimetric assays [24,25]. Silver nanoparticles have received more attention from researchers over time because of their size and shape-dependent distinctive properties. The developments in science with time have been enabling the conversion of the bulk form of silver to silver nanoparticles (AgNPs). Silver (Ag) is relatively cheap and eco-friendly and the plasmonic enhancement factor for silver in spectroscopic measurements is also higher [26]. Moreover, AgNPs are better antibacterial agents [27,28] due to their higher surface area to volume ratio in contact with bacteria [29]. AgNPs have been found to be potential antimicrobial agents against Gram-negative and Gram-positive bacteria [30,31,32].

Previously, synthesized AgNPs have been found to interact with mercury, leading to the detection of Hg^2+^ in the water [33,34,35]. The diameter and interactions of the AgNPs are two advantageous factors in designing modified AgNPs with improved mercury detection and removal capability [36]. Carboxylic acid-capped AgNPs show better antimicrobial properties even against drug-resistant strains [37,38,39,40]. As-synthesized silver nanoparticles either capped with hydrophilic ligands or carboxylic acids have also been reported to detect Hg+ at ppm levels [33,41]. The nanoparticles with different carboxylic acids on their surface have been characterized previously, where the NPs capped with adipic acid were comparatively more uniform and mono-disperse, hence suitable for biological applications [42]. Our research goal was to prepare AgNPs capped with adipic acid (AgNPs@AA). These AgNPs@AA were probed as the colorimetric sensors for Hg^2+^ detection and antimicrobial potential. To our knowledge, this is the first report about the detection of Hg^2+^ by AgNPs AA with the least LOD compared with previously reported AgNPs capped with other carboxylic acids [41,43].

## 2. Results

### 2.1. Synthesis and Characterization of Adipic Acid-Capped Silver Nanoparticles

**Figure 1:**
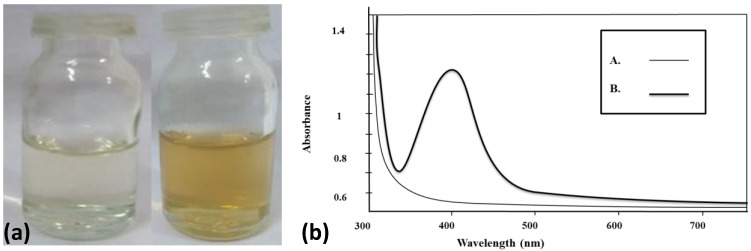
(**a**) Visual color change for the formation of AgNPs@AA (**b**) UV–Vis spectra of solution of adipic acid (A) and AgNPs@AA (B).

AgNPs@AA was prepared by reducing Ag^+^ using NaBH_4_, followed by capping AgNPs with adipic acid. The change in color of the reaction mixture from colorless to light yellow after adding a few drops of NaBH_4_ indicates the AgNPs@AA synthesis. The silver ions (Ag^+1^) are reduced to Ag^0^, which are then combined to form AgNPs, evident by a color change from colorless to light yellow [44], as shown in Figure 1a. To determine the concentration of AgNPs@AA, the number of Ag atoms in one NP of AgNPs@AA was found to be 245.1. The prepared AgNPs@AA concentration was about 0.11 nM (calculations are shown in Supplementary Materials).

The AgNPs@AA were synthesized by reducing AgNO_3_ with NaBH_4_ in the presence of adipic acid. By the action of reducing agent NaBH_4_, nucleation occurred, and silver ions/adipic acid complexes turned into AgNPs. Adipic acid is a dicarboxylic acid that possesses the ability to resonate a lone pair of electrons on oxygen with the carbonyl. It is well established that the carboxylate group (—COO^−^) is a functional group that is commonly used to stabilize silver nanoparticles [45,46].

The carboxylate moieties of adipic acid were adsorbed on the silver surface of AgNPs@AA with a bi-dentate bridging arrangement. The —COO^−^ moieties are bound to silver (sp orbital) via two oxygen atoms of the carboxylate moieties.

The successful synthesis of AgNPs@AA can also be confirmed by comparing the UV–Vis spectra of adipic acid and AgNPs. The UV–Vis spectra of adipic acid and AgNPs@AA are presented in Figure 1b. No absorption band was observed in the adipic acid spectrum, while the spectrum of AgNPs@AA has a characteristics surface plasmon resonance (SPR) absorption peak at 404 nm. AgNPs show a UV–Visible absorption maximum in the range of 390–490 nm [47,48]. The well-defined SPR absorption band and narrow width showed that prepared AgNPs were of narrow size distribution.

**Figure 2:**
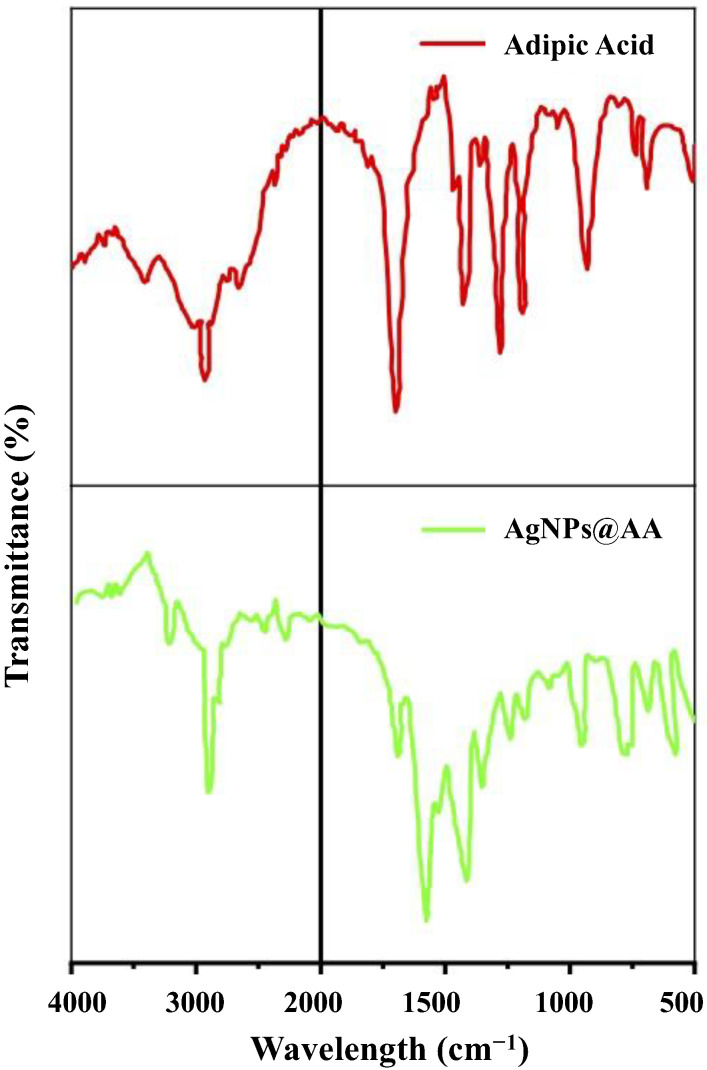
FTIR Spectra of adipic acid and AgNPs@AA.

FTIR spectroscopic analysis classifies chemical compounds in a wide range of capacities. FTIR is operative for detecting functional groups, which pave the way for identifying capping, stabilizing, or reducing agents for nanoparticles [49]. FTIR confirmed the successful capping of AgNPs@AA by adipic acid. The FTIR spectra of adipic acid and AgNPs@AA are shown in Figure 2, and all characteristic peaks are listed in Table S2. In the spectrum of adipic acid alone, the specific vibrations of adipic acid are seen at wavelength 3400 cm^−1^ because of the existence of OH, 2954 cm^−1^ attributable to stretching of C-H bond, 1697 cm^−1^ assigned to C=O symmetrical stretching, 1195 cm^−1^ due to C-O bond [50]. Comparison of spectra of adipic acid and AgNPs@AA showed that all the peaks present in the adipic acid spectrum were also present in the spectra of AgNPs@AA with a little change in peak position (from 3400 cm^−1^ to 3365 cm^−1^ and from 1697 cm^−1^ to 1685 cm^−1^). This retention of peaks in the spectrum of AgNPs@AA confirms the successful capping of AgNPs by adipic acid.

The AgNPs@AA were synthesized by reducing AgNO_3_ with NaBH_4_ in the presence of adipic acid. Adipic acid is a dicarboxylic acid that possesses the ability to resonate a lone pair of electrons on oxygen with the carbonyl. It is well established that the carboxylate group (—COO^−^) is a functional group that is commonly used to stabilize silver nanoparticles [45,46]. The carboxylate moieties of adipic acid were adsorbed on the silver surface of AgNPs@AA with a bi-dentate bridging arrangement. The —COO^−^ moieties are bound to silver (sp orbital) via two oxygen atoms of the carboxylate moieties, as evidenced by the peak present at 1573 cm^−1^ for the carboxylate group and peaks in the fingerprint region at 513 cm^−1^ and 603 cm^−1^ for bending vibrations of O-Ag in the FTIR spectrum of the AgNPs@AA surface [51]. These results indicate that the surface of AgNPs was capped by adipic acid.

### 2.2. SEM and XRD Measurement

**Figure 3:**
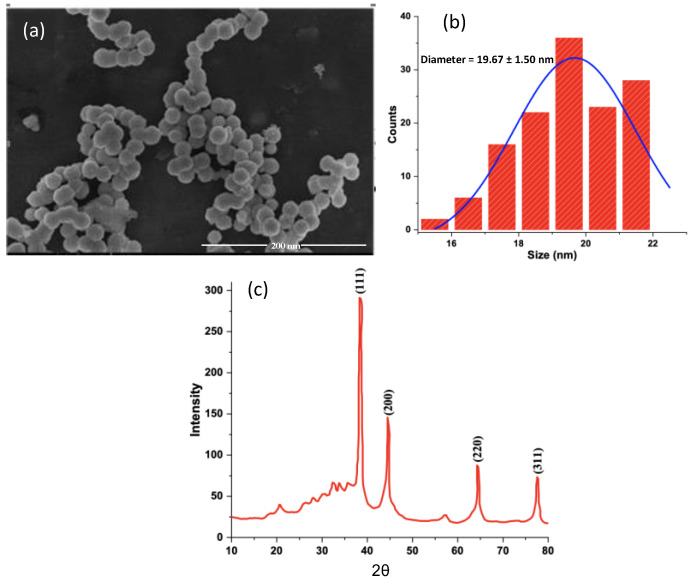
(**a**) SEM image of AgNPs@AA, (**b**) histogram for the size distribution of AgNPs@AA estimated from SEM image, and (**c**) XRD pattern of AgNPs@AA.

SEM images were recorded to examine the particles’ shape and size. Figure 3a displays an SEM image of the prepared NPs. This image shows a narrow particle size distribution and almost uniform spherical appearance. Analysis of the particle sizes revealed an average diameter of about 19.7 ± 1.5 nm, which is different from the value obtained from DLS (30.0 ± 5.0 nm). Note that the DLS size of the AgNPs@AA is greater than that measured from SEM images (19.7 ± 1.5 nm). The difference is due to the nature of the two measurements, where DLS measures the hydrodynamic size, including the solvation shell, and during the sample drying for SEM measurements, slight shrinkage might occur.

XRD analysis of the AgNPs@AA was carried out to confirm their crystalline nature. The XRD pattern of AgNPs@AA exhibited characteristic Bragg peaks of silver nanocrystals and is presented in Figure 3. The peaks observed at 2θ values 38.3°, 44.4°, 64.4°, and 77.6° were linked to different diffraction lattices planes of 111, 200, 220, and 311, respectively. This set of lattices planes confirmed the face centered cubic structure of AgNPs@AA (JCPDS, File No. 04-0783). 

The Debye–Scherer equation (Equation (1)) was used to assess the average AgNPs@AA size.

(1) $$D=\frac{K\lambda }{\beta Cos\theta }$$

where *D* is average nanoparticle size, and *K* is Scherer constant with a value of 0.94 for spherical crystallite size with cubic symmetry. *λ* is the wavelength of X-ray used for diffraction, i.e., Cuκα = 0.154 nm. ‘*β*’ is full width at half maximum (FWHM), and ‘*θ*’ is the diffraction angle. The XRD intensity for the peak at 38.29° was taken into account to calculate size. The values of *β*—i.e., FWHM in radian—were found to be 0.00939 at 2θ values of 38.29°. The average particle size using the Debye–Scherrer equation was calculated to be 19.5 nm. The size of AgNPs obtained from XRD studies was in agreement with that measured from SEM analysis (19.7 ± 1.5 nm) (see Figure 3).

**Figure 4:**
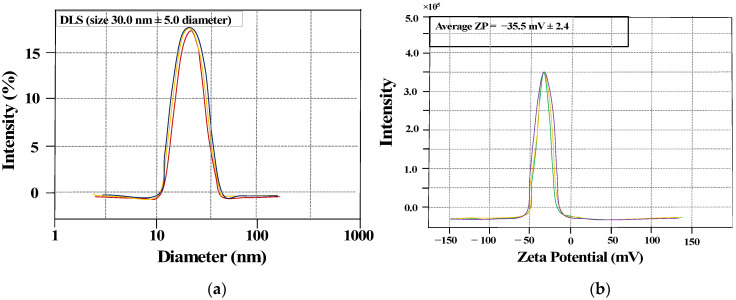
DLS analysis of AgNPs@AA having a diameter of 30 nm (**a**) Zeta potential of AgNPs@AA (**b**).

DLS and ZP measurement plots are shown in Figure 4. The average hydrodynamic particle size distribution determined for AgNPs@AA by dynamic light scattering (DLS) was approximately 30 ± 5.0 nm (Figure 4a). However, the zeta potential value of synthesized silver nanoparticles was −35.5 ± 2.4 mV (Figure 4b). The surface of AgNPs@AA was negatively charged due to the presence of carboxylate ions. The high value of ZP indicates that AgNPs@AA particles are electrostatically stabilized [52].

### 2.3. Antibacterial Activity

The adipic acid showed negligible activity against all bacterial strains, while AgNPs showed good antibacterial activity against *E. coli*, *B. subtilis*, *S. typhi*, and *S. aureus*. The AgNPs@AA showed promising antibacterial activity against all four clinically isolated bacteria—i.e., *E. coli*, *B. subtilis*, *S. typhi*, and *S. aureus* (Table 1). The AgNPs@AA seems to be more potent against *S. aureus* showing a zone of inhibition (ZoI) of 26.1 ± 0.7 mm with a MIC of 5.1 µg/mL. Interestingly, AgNPs@AA have shown considerable antimicrobial activity against XDR *S. typhi* strain, showing a zone of inhibition of 17.7 mm and a MIC of 16.2 µg/mL (Table 1). However, AgNPs without capping did not show considerable antimicrobial activity against XDR *S. typhi* strain (Table 1).

AgNPs have been suggested as solid antimicrobial agents [53,54,55]. The size and formulations of AgNPs play an important role in imparting antibacterial properties [56]. AgNPs have shown strong bacterial inhibitory growth potential against antibiotic-resistant bacteria [57]. Organic acids—i.e., adipic acid in their form—show strong antibacterial potential [58], and their presence in the antibiotic and hydrogel formulations augments their individual antibacterial activity [59,60]. Previously, the reduced AgNPs have shown strong antibacterial properties against animal-isolated multidrug-resistant (MDR) Salmonella strains [61]. Here, AgNPs capped with adipic acid, AgNPs@AA are shown to have better antibacterial potential as expected (Table 1). These have shown considerable antibacterial activity against both Gram-positive and Gram-negative bacteria. The growth of clinically isolated XDR *S. typhi* was inhibited far better than the standard drug, cefixime (Table 1). The cytotoxicity of AgNPs has already been probed in many studies, and it was observed that AgNPs can induce cytotoxicity in mammalian cells at higher concentrations. Here, it was found that the concentration of AgNPs@AA showing antimicrobial activities is less toxic than previously reported carboxylic acid-capped NPs (unpublished data). This indicates that further studies can explore the potential of AgNPs@AA against antibiotic-resistant bacteria and that they can be employed.

### 2.4. Detection of Hg^2+^ Ions by AgNPs@AA

**Figure 5:**
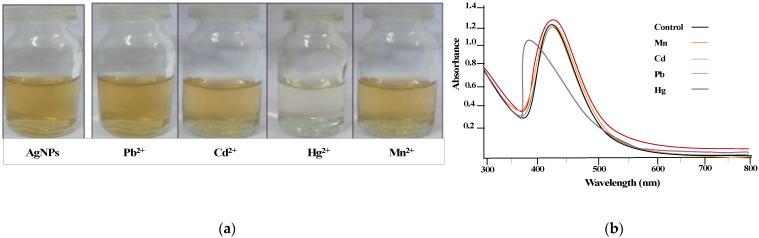
Visual response (**a**) and UV–Vis spectra (**b**) of different metal ion interactions with AgNPs@AA.

The potential of synthesized AgNPs@AA for selective detection of heavy metal ions in an aqueous solution was investigated by interacting different metal ion solutions (Pb^2+^, Cd^2+^, Mn^2+^, Hg^2+^) with AgNPs@AA. There was no change in color when Pb^2+^, Cd^2+^, or Mn^2+^ solution was added to AgNPs@AA suspension. A significant color change, light yellow to colorless, was observed when Hg^2+^ interacted with AgNPs@AA suspension, as shown in Figure 5a. The UV–Vis spectrum showed a noticeable change in the characteristic SPR peak upon the interaction of Hg^2+^ with AgNPs@AA, as shown in Figure 5b. Such changes were not observed upon interaction with other metal ions (see Figure 5b). This color change was due to the reduction of Hg^2+^ to Hg^0^ by AgNPs@AA (Ag^0^ converted to Ag^+1^). The mechanism of mercury ion detection by AgNPs@AA can be explained based on the reduction potential values of Ag^+^ and Hg^2+^ ions [62]. The standard reduction potentials for mercury and silver are +0.92 V and +0.80 V, respectively, and metals with higher reduction potential act as oxidizing agents [63]. These freshly generated Hg atoms give a blue shift by diffusion into the silver surface and decrease the characteristic absorption band due to substantial color change. Similarly, in a previous study, AgNPs aggregates were formed because of the diffusion of Hg atoms [64].

### 2.5. Systematic Performance of the Optical Sensor for the Detection of Hg^2+^

**Figure 6:**
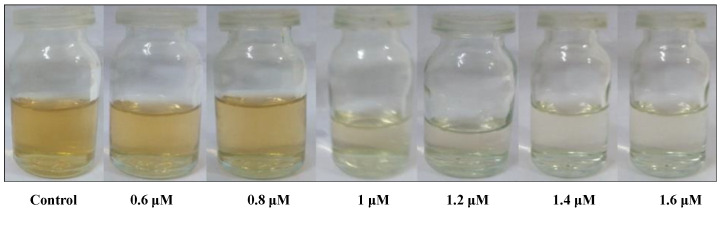
An in situ colorimetric assay for Hg^+2^ detection by the naked eye.

**Figure 7:**
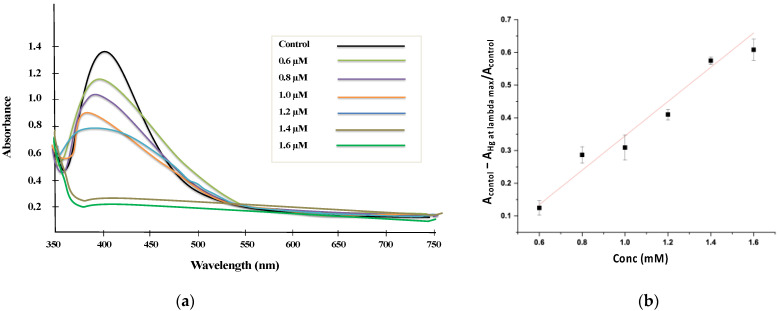
UV–Vis spectra (**a**) and Calibration curve (**b**) for the interaction of various concentrations of Hg^+2^ ions.

The limit of detection (LOD) for Hg^2+^ was determined by characteristic SPR peak using UV–Vis spectra of as-synthesized AgNPs@AA. Figure 6 shows the in situ colorimetric assays for Hg^+2^ detection by the naked eye. As the concentration of Hg^2+^ increased, the absorbance peak of AgNPs@AA decreased. A linear line (y = 0.0976x + 0.2436) with R^2^ = 0.9933 was obtained when the varying concentrations of Hg^2+^ were taken as abscissa and the absorbance ratio (A_Control_ − A_Hg_/A_Control_) as ordinate in the concentration range from 0.6–1.6 μM (Figure 7). A LOD of 0.12 μM was obtained for mercury detection by AgNPs@AA using the following formula.

$$\mathrm{LOD}=\frac{3\left(\mathrm{Standard}\;\mathrm{deviation}\;\mathrm{of}\;\mathrm{slope}\right)}{\mathrm{Slope}\;\mathrm{of}\;\mathrm{the}\;\mathrm{calibration}\;\mathrm{curve}}$$

Figure 7 shows that increasing the concentration of Hg^2+^ ions might cause a blue shift on the surface plasmon absorption band. A colorless solution was obtained by increasing the Hg^2+^ concentration. When a small concentration of Hg^2+^ was used, fewer Hg^2+^ ions were reduced to Hg. Hence, fading of the yellow color was very slight. As the concentration of Hg^2+^ ions increases, colloidal mercury drops due to an increase in reduced Hg. Therefore, a significant change in color from light yellow to colorless was observed by increasing the Hg^2+^ ions concentration in the prepared samples.

Table 2 includes the LOD and linear ranges for detecting Hg^2+^ using AgNPs stabilized by different methods as a colorimetric sensing probe. It is evident from the literature that the present method occupies a prominent place among those of earlier reported colorimetric AgNPs-based sensors [65].

The scientific reason why the AgNPs@AA interaction with other metal ions does not result in any color change can be explained based on their reduction potential values. Pb(II), Cd(II), and Mn(II) have potential reduction values of −0.13 V, −0.41 V, and −1.18 V, respectively. These values are lower than Ag (+0.80), so these metal ions cannot oxidize Ag atoms present in silver nanoparticles. Meanwhile, Hg(II) has a higher reduction potential value (+0.92 V) than Ag(I), which spontaneously oxidizes Ag atoms to Ag(I), turning the solution from yellowish to colorless. Then the Ag(I) ions react with Hg(II), leading to Ag-Hg nanoalloy formation. This redox reaction provides the basis of all such reactions in which AgNPs are used for colorimetric detection of Hg(II) [70]. Another reason for this selective detection of Hg(II) by AgNPs@AA is the strong binding energies of Hg(II) (5d^10^) with Ag(I) (4d^10^) based on the high-affinity metallophilic interaction, which causes the aggregation of AgNPs due to the metallophilic bonding between the d^10^ center having similar interactions [71,72].

### 2.6. Effect of Response Time on Colorimetric Assay

In order to investigate the response time of the nano-probe, 1.6 µM solution of the Hg^2+^ was added to the AgNPs@AA suspension, and the SPR peak was monitored for 10 min under the optimum conditions. This time-dependent spectral response was recorded after every 30 s. An immediate drop in the SPR peak intensity was witnessed. After 4 min, the SPR peak intensity reached a relatively constant value and did not show a significant decrease. The same outcome was achieved when the color change of the AgNPs@AA was visualized by the naked eye. The suspension became almost colorless after 4 min because the oxidation of AgNPs was almost completed within 6 min. This rapid response time makes this method an effective nano-probe for Hg^2+^ detection in real-time analyses (Figure S1a).

### 2.7. Effect of pH and Temperature on Colorimetric Assay

Synthesis and stabilization of the capped nanomaterial are controlled by the pH of the medium. The change in pH of the medium might change the electrical charges of the capping agent, which might result in the growth of the nanoparticles (Priya et al., 2016). To assess the environmental acceptance of the AgNPs@AA as a colorimetric nano-probe, the effect of pH on the stability of AgNPs was investigated. For this purpose, colorimetric detection of Hg^2+^ by AgNPs@AA suspension containing 0.6 µM Hg^2+^ was carried out within a pH range 3–9 (at higher pH, Hg^2+^ is precipitated as Hg(OH)_2_). The efficiency of the AgNPs@AA for the sensing of Hg^2+^ was almost unaffected throughout this pH range of 3–9, as shown in Figure 1b. These results reveal that these AgNPs@AA can be successfully employed to detect Hg^2+^ over a wide pH range.

To investigate the effect of temperature on the colorimetric detection of Hg^2+^ by AgNPs@AA, suspension of AgNPs@AA containing 0.6 µM Hg^2+^ was maintained at a different temperature from 25 to 55 °C. The efficiency of the AgNPs@AA for the sensing of Hg^2+^ increased slightly with an increase in temperature from 25 °C to 40 °C, and then a little decrease was observed, as shown in Figure S1c. An effective detection was observed at 40 °C. 

### 2.8. Real Sample Analysis

Finally, the potential of the proposed nano-probe for the detection of Hg^2+^ in natural water samples (river and tap) was also investigated. The real water samples were collected from river Jhelum District Sargodha and tap water from the chemistry laboratory, University of Sargodha, Sargodha. Different concentrations of Hg were added to these actual water samples. Recovery and the relative standard deviation (RSDs) of real water samples are summarized in Table S1. The recovery percentages of spiked water were found to be greater than 93%, with an RSD value of about 4%. These results show that this colorimetric nano-probe could be a reliable and effective method for Hg detection in real water samples.

## 3. Materials and Methods

The chemicals used in the study included hexanedioic acid or adipic acid (C_6_H_10_O_4_), silver nitrate (AgNO_3_), mercuric chloride (HgCl_2_), sodium borohydride (NaBH_4_), manganese nitrate (Mn (NO_3_)_2_.4H_2_O), cadmium nitrate (Cd (NO_3_)_2_), and lead nitrate (Pb (NO3)_2_).

### 3.1. Preparation of Adipic Acid-Capped Silver Nanoparticles

AgNPs@AA were prepared by reducing Ag^+^ from AgNO_3_ with a reducing agent NaBH_4_ followed by capping with adipic acid. The AgNO_3_ (3.38 mg, 1 mM, 20 mL) and adipic acid (2.9 mg, 1 mM, 20 mL) solutions were mixed in distilled water under vigorous magnetic stirring. Then NaBH_4_ (2.2 mg, 1 mM, 60 mL) solution in distilled water was added dropwise to the above mixture. After adding a few drops of NaBH_4_, the color of the solution changed from colorless to light yellow, confirming the formation of AgNPs@AA. The suspension so obtained was then centrifuged at 6000 rpm for 30 min. The supernatant was decanted, leaving pure silver nanoparticles at the bottom of the centrifugation tube. The as-obtained AgNPs@AA were centrifuged again after washing with distilled water. This washing process was repeated several times to remove any unreacted material. A part of these pure AgNPs@AA, obtained after washing, was stored as a solid sample to carry out different analyses. At the same time, the remaining fraction was sonicated after adding distilled water to obtain a suspension of pure AgNPs@AA. This suspension obtained was used as a stock solution of AgNPs@AA for further experiments. The concentration of the stock solution of AgNPs@AA was measured by a method reported earlier with the help of the UV–Vis technique (See Supplementary Materials) [73,74,75].

### 3.2. Characterization of AgNPs@AA

To confirm the successful synthesis of AgNPs@AA, UV–Vis spectra of adipic acid solution and AgNPs@AA suspension were recorded within 300–800 nm using a UV–Vis spectrophotometer (UV-1800 spectrophotometer, Shimadzu, Kyoto, Japan). FTIR spectra of synthesized AgNPs@AA and adipic acid were recorded in KBr pellets in the range of 600–4000 cm^−1^ to confirm the success of capping the AgNPs with adipic acid.

Zeta potential (ZP) and hydrodynamic size of AgNPs@AA were measured by using the Nano Zeta Sizer (ZS) system (Malvern Instruments, Malvern, UK). Different constraints of measurements were as follows: scattering angle (173°), laser wavelength (633 nm), temperature (25 °C), the refractive index of the medium (1.330), the refractive index of the material (0.200), and viscosity of medium (0.8872 mPa.s). For DLS measurements, the sample was initially passed through membrane PVDF (polyvinylidene fluoride) of 0.2 μm diameter. The DLS analyses were performed using normal resolution (general-purpose mode) and high resolution (with multiple narrow modes). The sample was put in a micro-cuvette made up of quartz, and results were recorded. The SEM analyses of AgNPs@AA were performed using an electron microscope (Nova, NanoSEM 450, Thermo Fisher Scientific, Hillsboro, OR, USA) furnished with a low energy detector named Everhart–Thornley detector after drying the nanoparticles over aluminum stubs.

The AgNPs@AA sample was also characterized by X-ray diffraction (XRD) to determine the crystal structure and size of the nanoparticle (Philips X’Pert-Pro X-ray diffractometer system, Malvern Panalytical, Malvern, UK). The X-ray tube was operated at a voltage of 40 kV, while the beam current was 30 mA. The 2θ range of the XRD patterns was between 10 and 80° under Cuκα = 0.154 nm. Scan speed during the analysis was kept at 5°/min.

### 3.3. Antibacterial Activity

The antibacterial activities of prepared AgNPs@AA were performed using the agar well diffusion assay against four clinically isolated bacteria: *Escherichia coli*, *Bacillus subtilis*, *Salmonella typhi*, and *Staphylococcus aureus*. The *S. typhi* strain used in this study was previously identified as an XDR strain causing XDR typhoid in patients [76]. The LB nutrient agar (Oxoid) plates were prepared after autoclaving, and bacterial culture lawns were prepared [77,78]. AgNPs@AA suspension (20 µL) was loaded on sterile disks and placed in LB agar plates to incubate at 37 °C overnight. A disk of a standard drug cefixime (5 µg) was used as a control. The inhibition zones for the control and AgNPs@AA were measured. Minimum Inhibitory Concentration (MIC) was estimated by making the serial dilutions of AgNPs@AA and standard drug in LB broth [77,78]. An inoculum of 10^6^ CFU/mL was used for MIC inhibition, and MIC was determined using a previously reported method for AgNPs [53].

### 3.4. Colorimetric Detection of Hg^2+^

To investigate the potential of synthesized AgNPs@AA for selective detection of heavy metal ions in an aqueous solution, different metal ion solutions (Pb^2+^, Cd^2+^, Mn^2+^, Hg^2+^) were interacted with AgNPs@AA. Each metal solution (1 µM, 0. 5 mL) was added to AgNPs@AA suspension (3 mL) in separate test tubes. Then UV–Vis spectrum of each solution containing metal ions and AgNPs@AA was recorded. Any apparent change in color or UV–Vis spectra of AgNPs@AA after the addition of a metal ion might provide the basis for the selective detection of the corresponding metal ion. A significant color change was observed for Hg^2+^. In order to determine the minimum concentration of Hg^2+^ that can be determined using the AgNPs@AA, a calibration curve was constructed by interacting variable concentrations (0.6–1.6 mM, 0.5 mL each) of Hg^+2^ with AgNPs@AA (3 mL) and recording their spectra.

## 4. Conclusions

The novel AgNPs@AA were found to be potential antimicrobial agents and excellent sensing probes for metal ion detection. These nanoparticles were effectively used for the selective detection of mercury ions in an aqueous solution. The color of the AgNPs suspension changed from light yellow to colorless by the interactions of AgNPs with mercury ions. The characteristic absorption peak in UV–Vis spectra was blue shifted. A LOD value of 0.12 µM with a linear range of 0.6–1.6 µM was obtained. AgNPs@AA have shown strong antibacterial potential against XDR *S. typhi* strain, and these can be further studied as an effective treatment against drug-resistant bacterial agents.

## Figures and Tables

**Table 1:** Antimicrobial activities of AgNPs against clinically isolated bacterial strains.

	Zone of Inhibition (MM)				MIC (µG/ML)	
*Bacterial Strains*	AgNPs	AA	AgNPs@AA	Cefixime	AgNPs@AA	Cefixime
*B. subtilis*	16.8 ± 3.2	3.0 ± 2.1	20.1 ± 2.5	18.0 ± 1.1	4.5 ± 0.9	3.3 ± 0.8
*S. typhi*	3.0 ± 0.2	ND	17.7 ± 1.3	7.0 ± 3.2	16.2 ± 1.0	64.1 ± 0.4
*S. aureus*	20.8 ± 1.4	ND	26.1 ± 4.0	24.3 ± 2.1	5.1 ± 1.1	9.4 ± 0.5
*E. coli*	13.3 ± 1.0	5.0 ± 1.3	19.7 ± 2.4	27.1 ± 1.5	13.7 ± 0.8	5.4 ± 0.1

**Table 2:** Comparison of AgNPs stabilized by different methods as a colorimetric sensing probe for Hg^2+^ determination.

Surface Stabilizing Media	Linear Range (µM)	LOD (µM)	Reference
Soap-root plant-based AgNPs	10–100	2.2	[66]
Fast orange peel-mediated AgNPs	1.0–100	1.2	[67]
Garlic extract AgNPs	2.0–75.0	2.0	[68]
Green biomimetic AgNPs	0.1–1.0	0.13	[69]
Citrate and γ-aminobutyric acid stabilized AgNPs	5.0–35.0	2.4	[41]
Peptide-conjugated AgNPs	1.0–100	4.1	[69]
Adipic acid stabilized AgNPs	0.6–1.6	0.1	Present Work

## Data Availability

Not applicable.

## Supplementary Materials

The following supporting information can be downloaded at: https://www.mdpi.com/article/10.3390/molecules27113363/s1, Table S1: Analytical results for the detection of Hg^2+^ using AgNPs@AA in real samples; determination of concentration of synthesized AgNPs@AA. Table S2: Characteristics peaks of FTIR spectra of adipic acid and AgNPs@AA. Figure S1: Effect of response time (a), pH (b), temperature (c) on the Hg^2+^ detection in real-time analyses. Determination of Concentration of Synthesized AgNPs@AA.
