# Supplementary material for: Novel Carboxylic Acid-Capped Silver Nanoparticles as Antimicrobial and Colorimetric Sensing Agents
Source: Molecules. 2022 May 24;27(11):3363. doi: 10.3390/molecules27113363 (PMC9182355; doi:10.3390/molecules27113363)
Supplement: Supplementary file 1 [file molecules-27-03363-s001.zip › molecules-1633128-supplementary.pdf]

molecules

MDPI

Supplementary Information

Novel Carboxylic Acid-Capped Silver Nanoparticles as Antimicrobial and Colorimetric Sensing Agents

Muhammad Imran Irfan [1,2], Fareeha Amjad [2], Azhar Abbas [2,3,*], Muhammad Fayyaz ur Rehman [2,*], Fariha Kanwal [4], Muhammad Saeed [5], Sami Ullah [2] and Changrui Lu [1,*]

[1] Department of Chemistry, Chemical Engineering and Biotechnology, Donghua University, Shanghai 201620, China; imran.irfan@uos.edu.pk
[2] Institute of Chemistry, Faculty of Science, University of Sargodha, Sargodha 40100, Pakistan; fareeha351@gmail.com (F.A.); samichemist30@gmail.com (S.U.)
[3] Department of Chemistry, Government Ambala Muslim Graduate College, Sargodha 40100, Pakistan
[4] Med-X Research Institute, School of Biomedical Engineering, Shanghai Jiao Tong University, Shanghai 201620, China; farihakaanwal@gmail.com
[5] Department of Chemistry and Chemical Engineering, SBA School of Science and Engineering, Lahore University of Management Sciences (LUMS) Lahore 54792, Pakistan; muhammad.saeed@lums.edu.pk
* Correspondence: azharabbas73@yahoo.com (A.A.); muhammad.fayyaz@uos.edu.pk (M.F.u.R); crlu@dhu.edu.cn (C.L.)

(a)

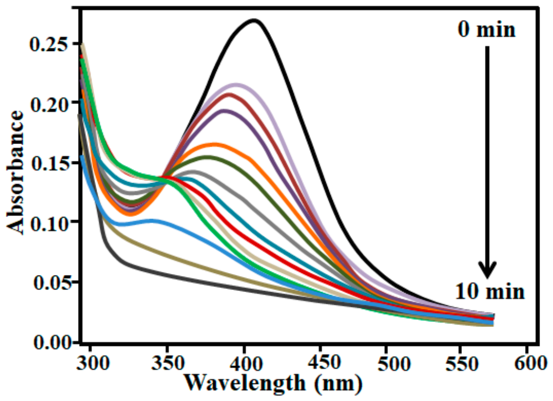

(b)

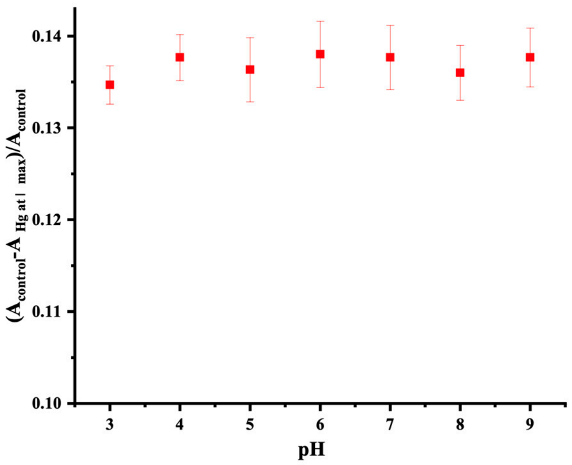

(c)

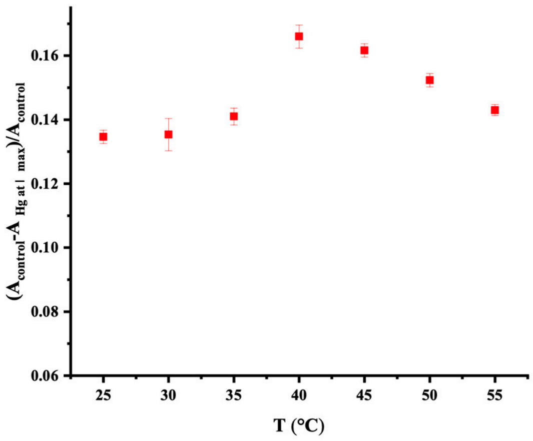

Figure S1. Effect of response time (A), pH (B), temperature (B) on the Hg^{2+}detection in real-time analyses.

Table S1. Analytical results for the detection of Hg^{2+} using AgNPs@AA in real samples.

Sample	Hg^{2+} Added (μM)	Hg^{2+} Found (μM)	Recovery (%)	RSD (%) *n* = 3
River	25	24.2	96.6	2.5
	50	49.1	98.2	5.2
	75	73.1	97.5	3.9
Tap	25	24.6	98.4	3.2
	50	47.6	95.3	4.1
	75	70.1	93.5	2.8

Table S2. Characteristics peaks of FTIR spectra of adipic acid and AgNPs@AA.

Band Origin	Wavenumber (cm^{-1})	
	**Adipic Acid**	**AgNPs@AA**
O-H Stretching	3400	3365
C-H Stretching	2954	2951
C=O stretching	1697	1685
COO^-	-	1573
C-O & C-H bending	1432	1418
C-O	1280 & 1198	1245 & 1175
CH deformation	1154	
C-C bond	925	961
C-H out of plane	746 & 690	788 & 682
O-Ag Bond		603 and 513

Determination of Concentration of Synthesized Ag NPs@AA

For the determination of concentration of Ag NPs@AA, the number of Ag atoms in one NP of Ag NPs@AA was found to be 245.10. The prepared Ag NPs@AA concentration was about 0. 11 nM.
The prepared Ag NPs@AA concentration can be calculated as below:

$$d = 20\,nm = 20 \times 10^{-9}\,nm$$

$$r = 10 \times 10^{-9}\,nm$$

$$V = \frac{4}{3}\pi\, r^3 \; = \frac{4}{3} \times 3.14 \times (10 \times 10^{-9}) = 4.19 \times 10^{-24}\,m^3$$

$$N_{A=6.022\times 10^{23}}$$

$$\rho = 10490\ g\ cm^{-3}$$

$$M = 107.868\ {}^{g}/_{mol}$$

$Number\ of\ silver\ atoms\ in\ one\ nanoparticle = (V * N_A * \rho)/M$ =245
Ɛ for AgNps can be calculated by equation

$$ln\text{Ɛ} = 1.4418 \ln D + 18.955$$

where D=Diameter of the Ag NPs
Ɛ was calculated by this equation for a particle of size 20 nm (measured from SEM)=1.28*10[10] $M^{-1}\ cm^{-1}$

$$A = 1.4$$

$$l = 10\,mm = 1\,cm$$

Concentration of AgNPs@AA

$$c = \frac{A}{\text{Ɛ} \times l} = \frac{1.4}{1.28 \times 10^{10} \times 1} = 1.1 \times 10^{-10}\,M = 0.11\,nM$$
